# Supplementary figures and images for: Variation in structural motifs within SARS-related coronavirus spike proteins
Source: PLoS Pathog. 2024 May 28;20(5):e1012158. doi: 10.1371/journal.ppat.1012158 (PMC11236199; doi:10.1371/journal.ppat.1012158)

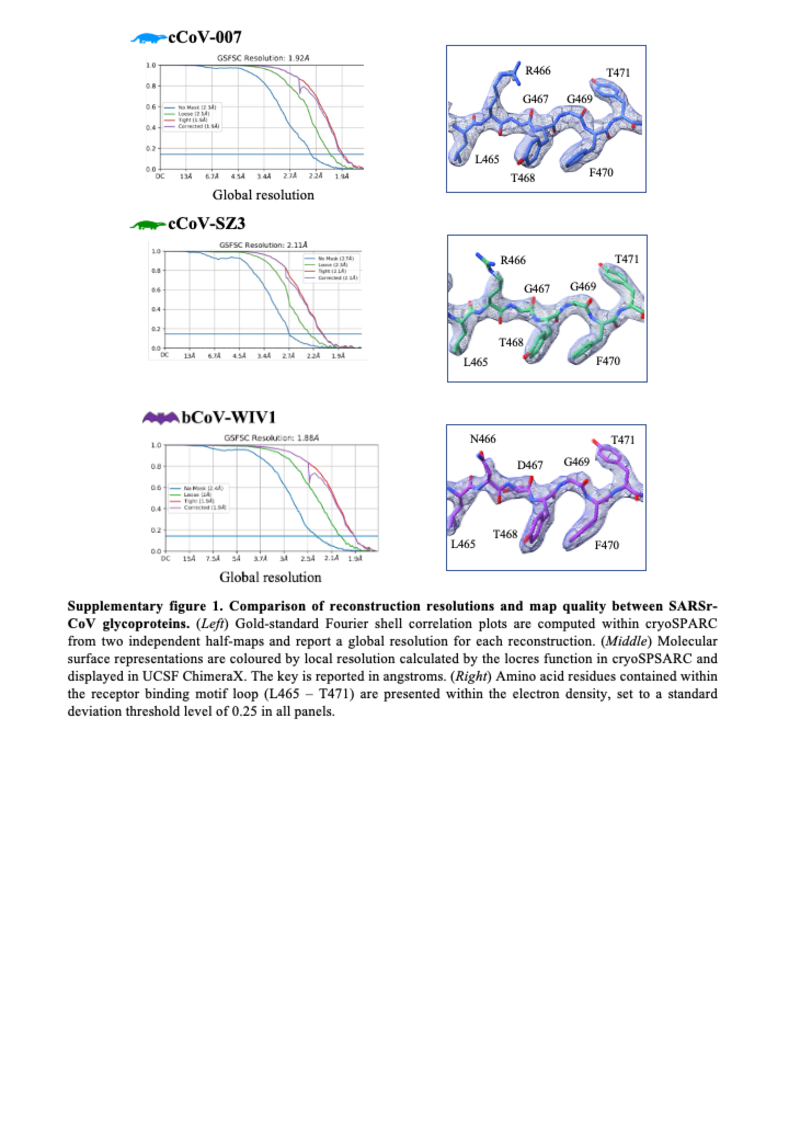

Supplement: S1 Fig — (Left) Gold-standard Fourier shell correlation plots are computed within cryoSPARC from two independent half-maps and report a global resolution for each reconstruction. (Right) Amino acid residues contained within the receptor binding motif loop (L465 –T471) are presented within the electron density, set to a standard deviation threshold level of 0.25 in all panels. (TIF) [file ppat.1012158.s001.tif]

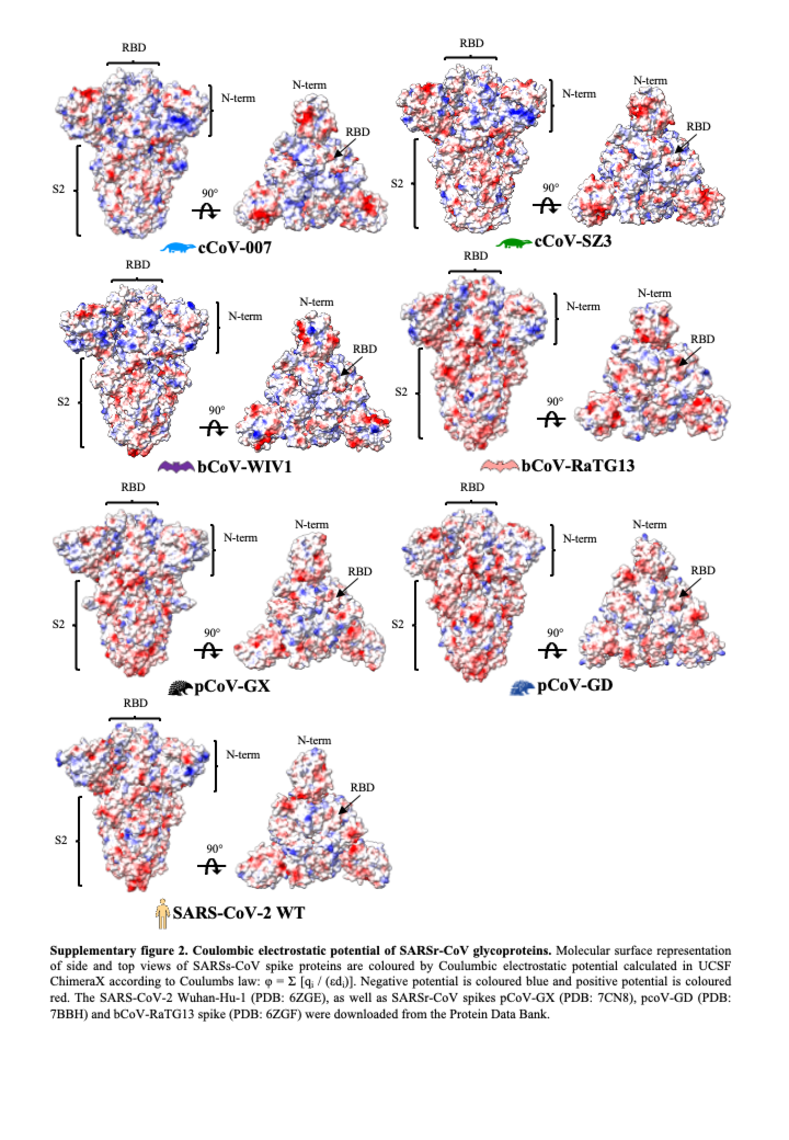

Supplement: S2 Fig — Molecular surface representation of side and top views of SARSs-CoV spike proteins are coloured by Coulumbic electrostatic potential calculated in UCSF ChimeraX according to Coulumbs law: φ = Σ [qi / (εdi)]. Negative potential is coloured blue and positive potential is coloured red. The SARS-CoV-2 Wuhan-Hu-1 (PDB: 6ZGE), as well as SARSr-CoV spikes pCoV-GX (PDB: 7CN8), pcoV-GD (PDB: 7BBH) and bCoV-RaTG13 spike (PDB: 6ZGF) were downloaded from the Protein Data Bank. (TIF) [file ppat.1012158.s002.tif]

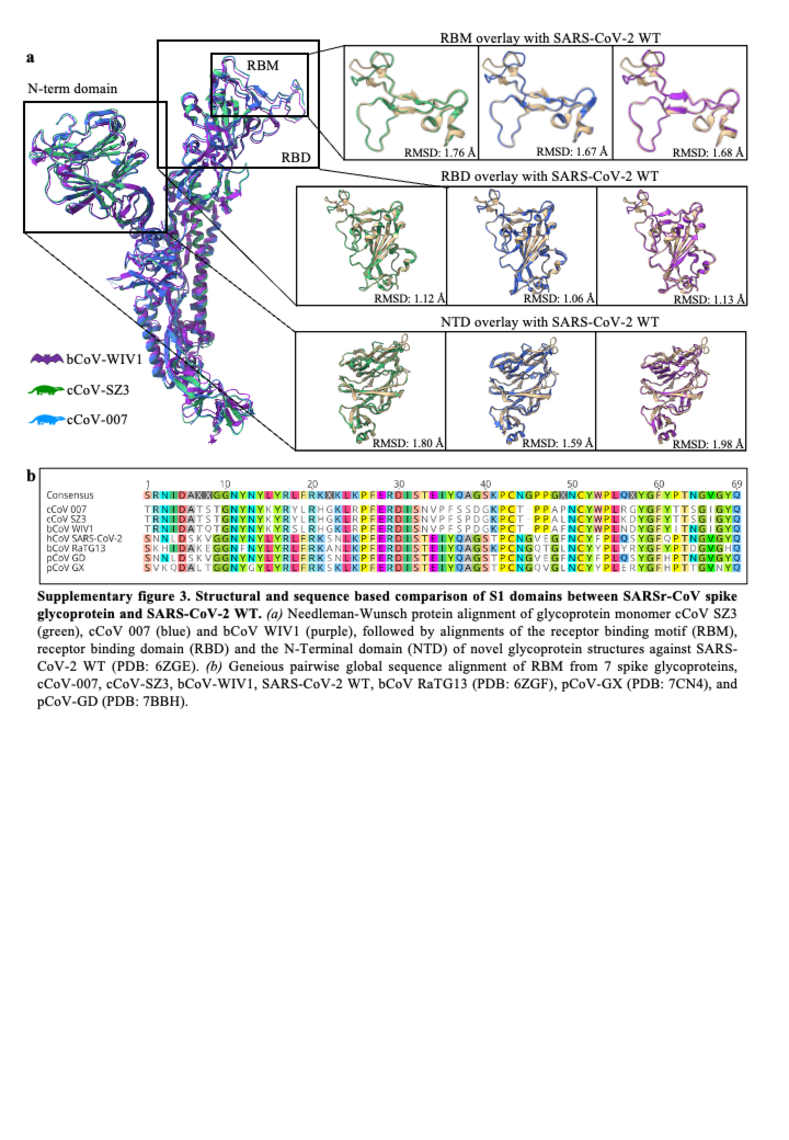

Supplement: S3 Fig — (a) Needleman-Wunsch protein alignment of glycoprotein monomer cCoV SZ3 (green), cCoV 007 (blue) and bCoV WIV1 (purple), followed by alignments of the receptor binding motif (RBM), receptor binding domain (RBD) and the N-Terminal domain (NTD) of novel glycoprotein structures against SARS-CoV-2 WT (PDB: 6ZGE). (b) Geneious pairwise global amino acid sequence alignment of RBM from 7 spike glycoproteins, cCoV-007, cCoV-SZ3, bCoV-WIV1, SARS-CoV-2 WT, bCoV RaTG13 (PDB: 6ZGF), pCoV-GX (PDB: 7CN4), and pCoV-GD (PDB: 7BBH). (TIF) [file ppat.1012158.s003.tif]

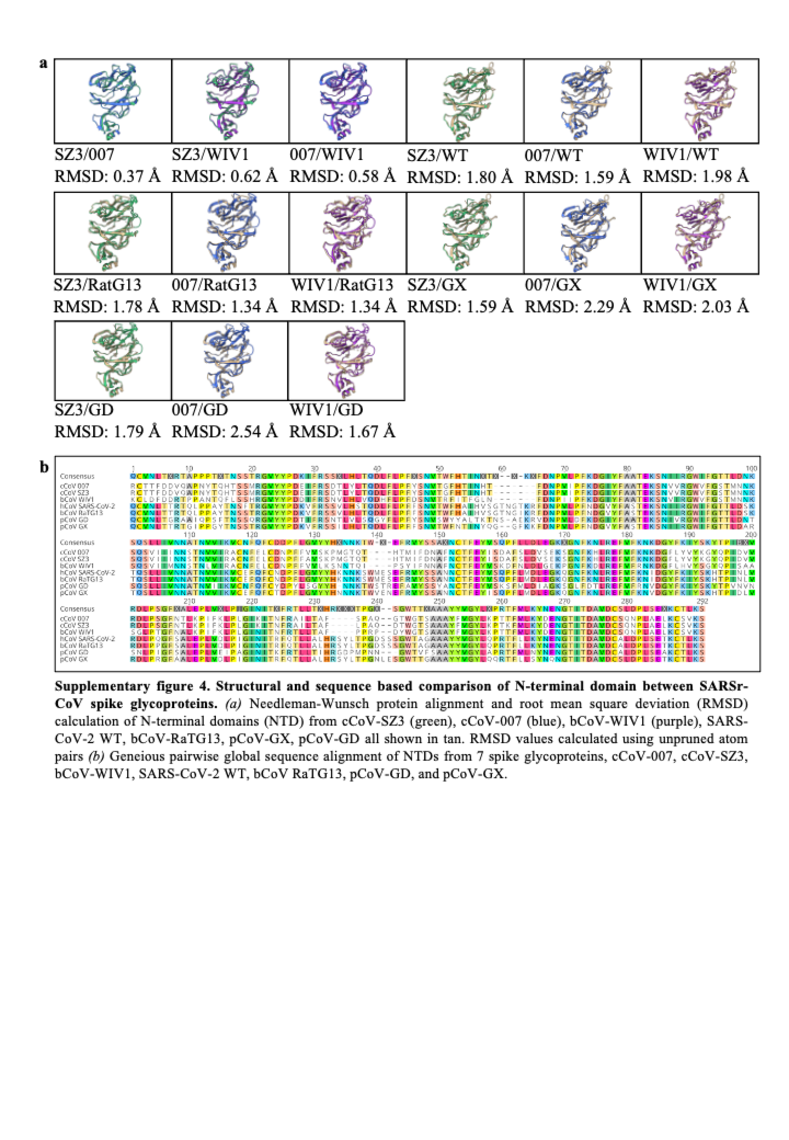

Supplement: S4 Fig — (a) Needleman-Wunsch protein alignment and root mean square deviation (RMSD) calculation of N-terminal domains (NTD) from cCoV-SZ3 (green), cCoV-007 (blue), bCoV-WIV1 (purple), SARS-CoV-2 WT, bCoV-RaTG13, pCoV-GX, pCoV-GD all shown in tan. RMSD values calculated using unpruned atom pairs (b) Geneious pairwise global amino acidsequence alignment of NTDs from 7 spike glycoproteins, cCoV-007, cCoV-SZ3, bCoV-WIV1, SARS-CoV-2 WT, bCoV RaTG13, pCoV-GD, and pCoV-GX. (TIF) [file ppat.1012158.s004.tif]

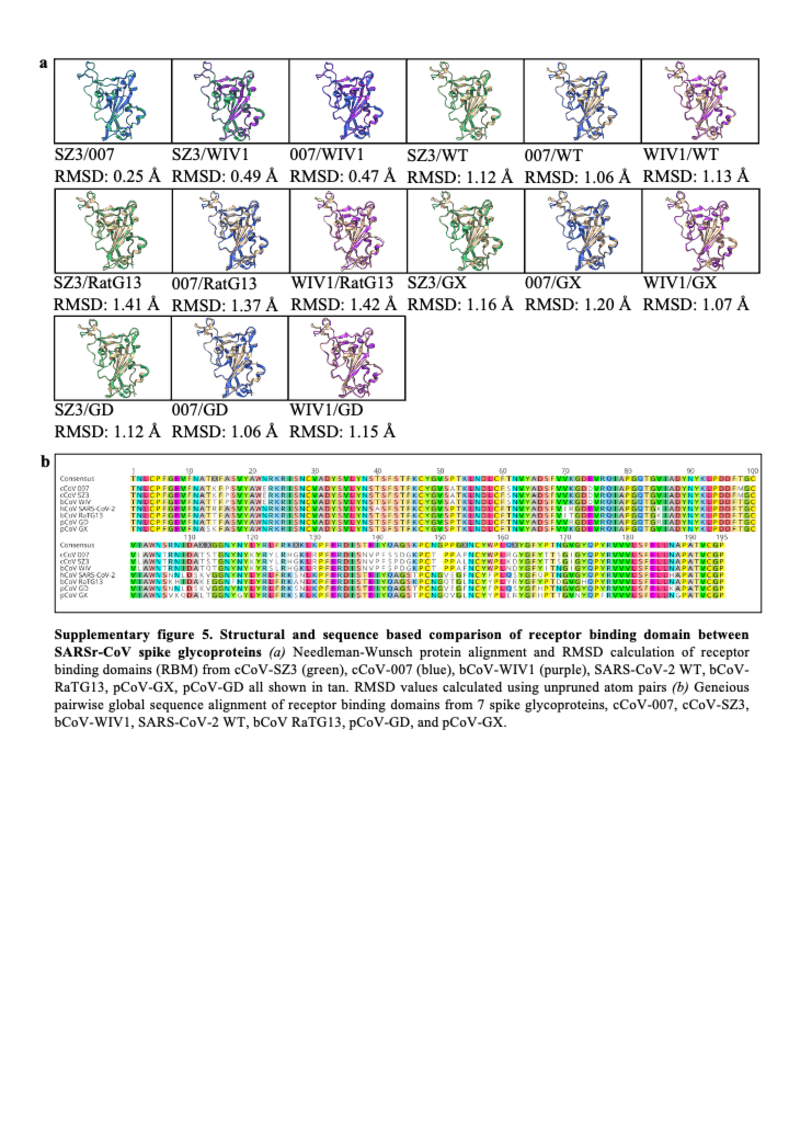

Supplement: S5 Fig — (a) Needleman-Wunsch protein alignment and RMSD calculation of receptor binding domains (RBM) from cCoV-SZ3 (green), cCoV-007 (blue), bCoV-WIV1 (purple), SARS-CoV-2 WT, bCoV-RaTG13, pCoV-GX, pCoV-GD all shown in tan. RMSD values calculated using unpruned atom pairs (b) Geneious pairwise global amino acid sequence alignment of receptor binding domains from 7 spike glycoproteins, cCoV-007, cCoV-SZ3, bCoV-WIV1, SARS-CoV-2 WT, bCoV RaTG13, pCoV-GD, and pCoV-GX. (TIF) [file ppat.1012158.s005.tif]

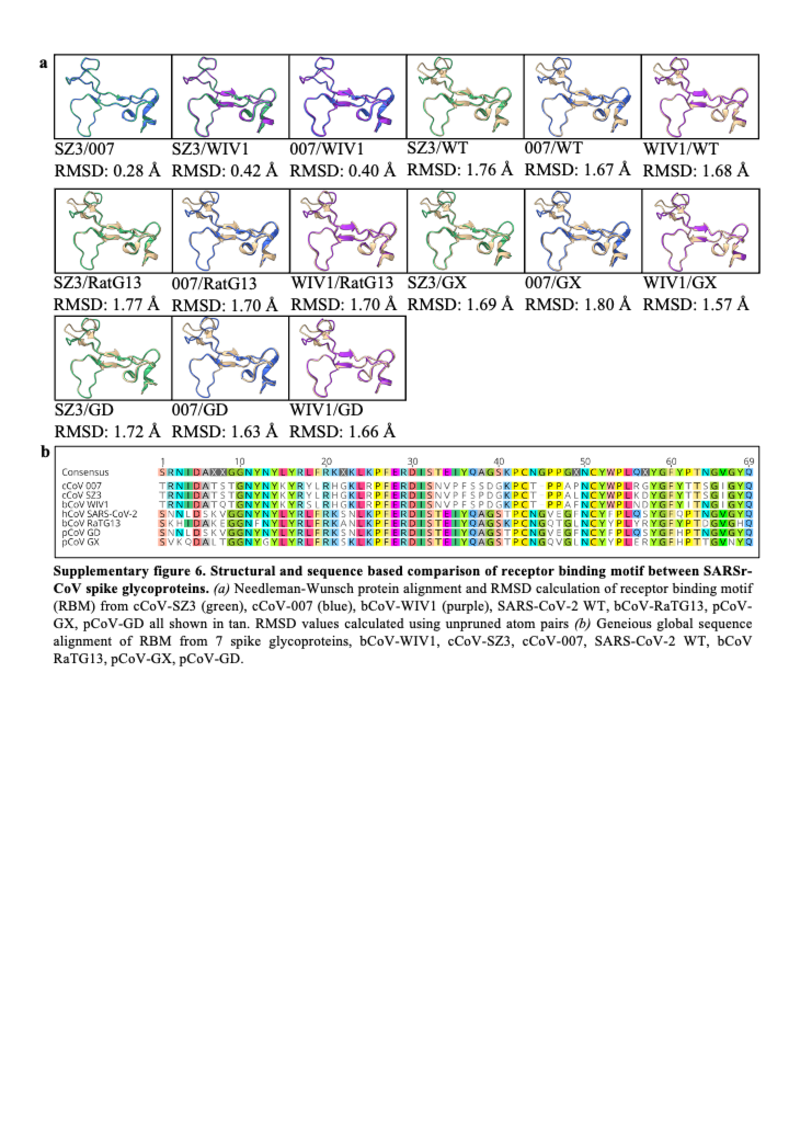

Supplement: S6 Fig — (a) Needleman-Wunsch protein alignment and RMSD calculation of receptor binding motif (RBM) from cCoV-SZ3 (green), cCoV-007 (blue), bCoV-WIV1 (purple), SARS-CoV-2 WT, bCoV-RaTG13, pCoV-GX, pCoV-GD all shown in tan. RMSD values calculated using unpruned atom pairs (b) Geneious global amino acid sequence alignment of RBM from 7 spike glycoproteins, bCoV-WIV1, cCoV-SZ3, cCoV-007, SARS-CoV-2 WT, bCoV RaTG13, pCoV-GX, pCoV-GD. (TIF) [file ppat.1012158.s006.tif]

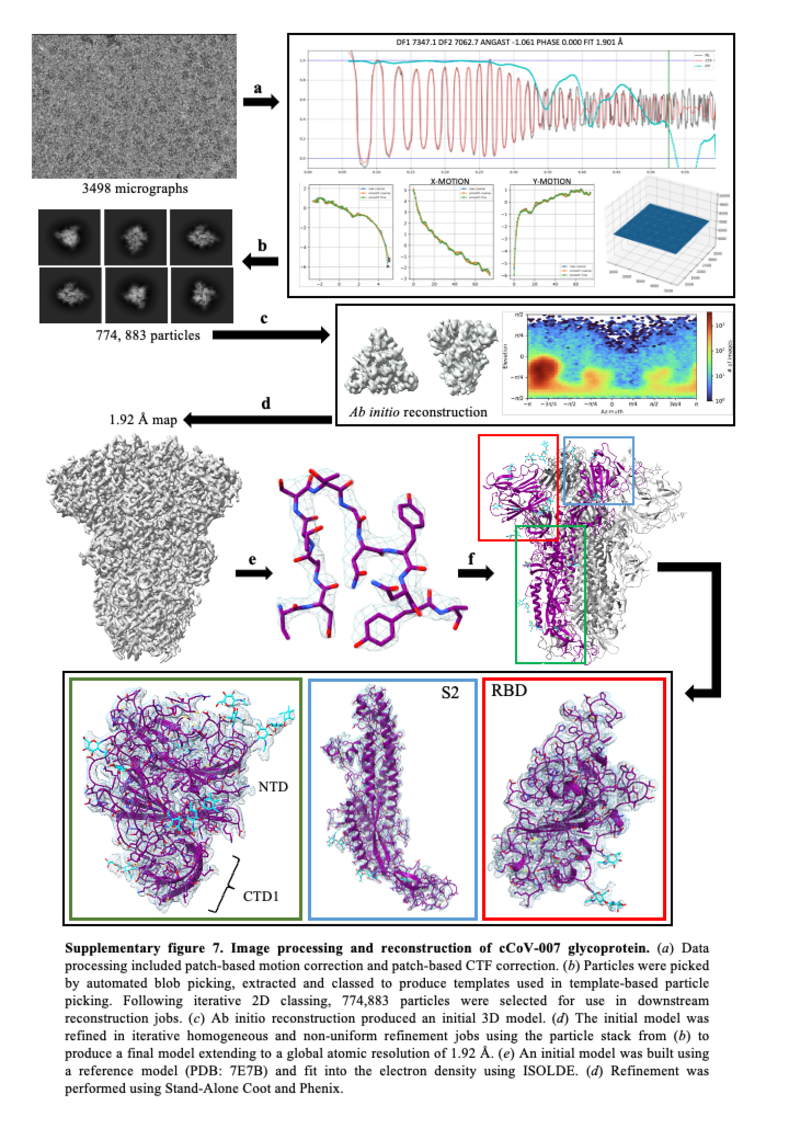

Supplement: S7 Fig — (a) Data processing included patch-based motion correction and patch-based CTF correction. (b) Particles were picked by automated blob picking, extracted and classed to produce templates used in template-based particle picking. Following iterative 2D classing, 774,883 particles were selected for use in downstream reconstruction jobs. (c) Ab initio reconstruction produced an initial 3D model. (d) The initial model was refined in iterative homogeneous and non-uniform refinement jobs using the particle stack from (b) to produce a final model extending to a global atomic resolution of 1.92 Å. (e) An initial model was built using a reference model (PDB: 7E7B) and fit into the electron density using ISOLDE. (d) Refinement was performed using Stand-Alone Coot and Phenix. (TIF) [file ppat.1012158.s007.tif]

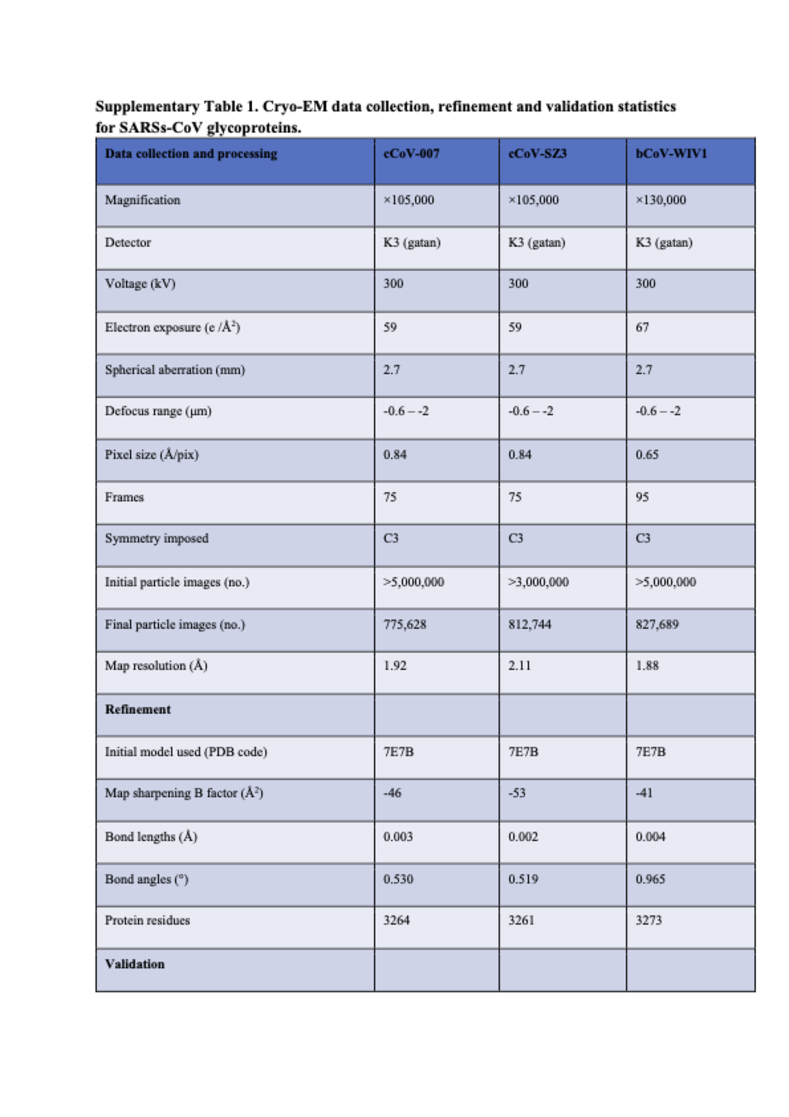

Supplement: S1 Table — (TIF) [file ppat.1012158.s008.tif]
