# Supplementary material for: Variation in structural motifs within SARS-related coronavirus spike proteins
Source: PLoS Pathog. 2024 May 28;20(5):e1012158. doi: 10.1371/journal.ppat.1012158 (PMC11236199; doi:10.1371/journal.ppat.1012158)
Supplement: Attachment — Submitted filename: ResponseToReviewers.docx [file ppat.1012158.s009.docx]

**PLOS Pathogens: Decision on your submission PPATHOGENS-D-24-00249 - [EMID:be607381c17537eb]**

We warmly thank both reviewers for their comprehensive review of our manuscript which we have worked very hard on. The paper is now much stronger thanks to the advice given by the reviewers and we are very grateful. Please see below our responses to the commentary provided, with edits attached where appropriate to make it easier for the reviewers to see what has been changed. Editor comments are in black and author comments are in green.

Reviewer #2:
Firstly, the introduction to the research background is excessively concise. It is recommended that the authors provide more detailed background information and explicitly state the research objectives.

We thank the reviewer for pointing this out and have made revisions to the introduction to provide more detailed background and explain the objectives of our research. We highlighted in yellow everything that was expanded in our edited revision submitted with these responses, but have not pasted it here for the purposes of brevity.

Secondly, there are numerous ambiguities and deficiencies in result interpretation, particularly regarding the inadequate validation of functional and stability hypotheses, which could significantly undermine the credibility and persuasiveness of the research.

We thank the reviewers for their insight. As we didn’t conduct any stability validation assays on our spikes and have analyzed the 3D structures in isolation, we hesitate to make strong conclusions about the function or stability properties of structural modifications without explicit supporting experimental data. We are therefore most comfortable with the speculative wording we made in our manuscript as it most closely represents our position.

Additionally, discrepancies between the descriptions in the text and figures, along with a lack of proper explanations of figure content within the text, make it challenging for readers to comprehend the research findings.

We have gone through our figure legends and everywhere in the body of the text where results are mentioned and have clarified the text where appropriate.

Finally, inconsistencies in capitalization and font color used in figures detract from overall formatting coherence of this paper. Standardizing these aspects will enhance readability and professionalism.

We have fixed this, where we were able to identify inconsistencies.

Reviewer #2: 1. In line 40, “Further, considering that three coronaviruses emerged zoonotically in the last……”, Which the three coronaviruses are referred to here?

We have edited this in the text to clarify we meant MERS, SARS, and SARS-CoV-2.

Further, considering that three coronaviruses emerged zoonotically in the last 20 years (MERS, SARS, and SARS-CoV-2), structural research into their closest genetic relatives is a matter of pandemic preparedness [5, 9].

2. In line 62, “As both bats and civets are potential intermediate hosts for SARS-CoV-2, this research narrows the structural knowledge gap on SARSr-CoV spikes, and informs future investigations into the origin of SARS-CoV-2.” There should be corresponding references cited here.

We have referenced this statement on line 79 and adjusted the wording to clarify that bats are in fact the natural host and civets are an intermediate host.

3. In line 69-72, “Several structural motifs were proposed to regulate this behavior in the case of SARS-CoV-2 spike” refers to which structural motifs? “We have chosen three SARSr-CoVs from……” , how were the three SARSr-CoV selected? The rationale for their selection is not clearly articulated.

We thank the reviewer for this comment. We have made mention of the structural motifs important for stabilisation of the ‘down’ conformation.

We believe we did articulate the selection rationale in the very first section of the discussion section, which we ended with the following sentence “These factors influenced our decision to select these three strains for structural study.” We have now made mention of this selection criteria briefly in the Introduction and the Results section as well. Updates:

Several structural motifs were proposed to regulate this behavior in the case of SARS-CoV-2 spike, including the D614 substitution, the fatty acid binding ability of the spike, and the trimer interface.

We have chosen three SARSr-CoVs from evolutionarily important hosts, namely of civet and bat origin given both these hosts are key suspects as intermediate hosts of SARS-CoV-2, in order to determine how structural variations in these motifs regulate stability of the glycoproteins.

4. In line 80, “WIV1 share the most structural similarity with SARS-CoV-2, with RMSD values 2.3 A, 2.0 A and 2.2 A”, The values 2.3 A, 2.0 A, and 2.2 A described are not found in the figures. WIV1 and SARS-CoV-2 are not the most similar, and the RMSD value does not indicate greater structural similarity. Instead, the larger the RMSD value, the less similar the structures are.

We have added a parentheses to this sentence to direct readers to the supplementary figure associated with this statement. We also noted that some of the RMSD values in this figure were indeed around the wrong way. As we fixed some of the C-beta outliers suggested below by the reviewers, we re-made all the RMSD panels with the updated RMSD. Based on domain-to-domain comparisons, 007 is the most similar to SARS-CoV-2. This is now reflected in the paper. We apologize and have corrected the figure appropriately.

5. In line 86,“Several non-conserved residues have altered the hydrogen bonding network that…….” Regarding the hydrogen bonding network described in the manuscript, it is not shown in the figure.

We believe we have shown the hydrogen bonding network of the scoop loop in panel B of figure 1. However, we have added a dashed line to the panel to indicate the distance shifted relative to each other. Thank you.

6. There are numerous inconsistencies between the Figures and the descriptions provided in the manuscript. Additionally, some Figures lack accompanying text descriptions. The order of Figures and text descriptions should be consistent.

Thank you for this great suggestion. We have clarified some of the wording in the text to expand and better describe the content of the figures. All our edits are in yellow within the text. Thank you.

Reviewer #1: 1. Lines 43-44 of the introduction: this appears to be a general statement about coronaviruses, yet it states that the receptor is ACE2, which is not used by all coronaviruses. This sentence should be reworded for accuracy.

We thank the reviewer for pointing out that we were not clear enough in this area. As we work only with SARS-related coronavirus, which only bind ACE2, we have clarified this. We are not referring to other coronaviruses (like feline or canine CoVs).

“SARSr-CoV spike proteins cover the coronavirus membrane, undergoing a suite of conformational changes to bind the viral receptor known as angiotensin converting enzyme 2 (ACE2) and mediate membrane fusion.”

2. Table S1: list the actual number of final particle images used in the reconstruction rather than an approximation.

We have updated this table appropriately.

3. Table S1: Under “refinement”, the row “Initial model used (PDB code)” lists “ab initio”, yet in the methods the authors state “Initial models were built by mutating a previously solved spike structure (PDB accession code: 7E7B)”. Therefore, 7E7B should be listed in this row of the table.

Thank you for noticing this. We have updated the table appropriately.

4. A value for the map-to-model resolution should be provided, rather than listing N/A

Thank you. We have removed the N/A and also added a Q score range to our table.

5. There is no reason for models to have C-beta outliers. These should be fixed and re-refined.

These have been fixed and the table updated appropriately.

6. PDB validation reports should be made available to reviewers to better assess the quality of the maps and models. This is now standard practice at many journals.

We apologize that the validation reports were not submitted at the time the manuscript was submitted. We will be sure not to do this again in the future. That was our oversight.

Reviewer #2: 1. The uppercase and lowercase letters in the figures should be consistent with those in the manuscript.

Thank you kindly for pointing this out! Both co-first authors participated in the writing of the manuscript, so there is some inconsistency because of the two different writers and their writing styles. We have fixed all the text references where appropriate.

2. Some words in the manuscript are black and some are dark gray.

We were unable to see where any text was gray… but have changed *all* font to a default black. All we can say is it was likely an issue with the colour management settings of our computer monitor.

Again, we’d like to reiterate our gratitude to the reviewers for their kind support of our manuscript and their attention to detail when critiquing our work. Thank you kindly.
